# Animal–chlorophyte photosymbioses: evolutionary origins and ecological diversity

**DOI:** 10.1098/rsbl.2025.0250

**Published:** 2025-07-30

**Authors:** Isabel Jiah-Yih Liao, Tosuke Sakagami, Thomas D. Lewin, Xavier Bailly, Mayuko Hamada, Yi-Jyun Luo

**Affiliations:** ^1^ Biodiversity Research Center, Academia Sinica, Taipei, Taiwan; ^2^ Laboratoire des Modèles Marins Multicellulaires, Station Biologique de Roscoff, Roscoff, Bretagne, France; ^3^ Ushimado Marine Institute, Okayama University, Setouchi, Okayama, Japan

**Keywords:** hydra, photosymbiosis, green algae, acoels, sponges

## Abstract

Photosynthetic symbiosis occurs across diverse animal lineages, including Porifera, Cnidaria, Xenacoelomorpha and Mollusca. These associations between animal hosts and photosynthetic algae often involve the exchange of essential macronutrients, supporting adaptation to a wide range of aquatic environments. A small yet taxonomically widespread subset of animals host photosymbionts from the core chlorophytes, a phylogenetically expansive clade of green algae. These rare instances of ‘plant-like’ animals have arisen independently across distantly related lineages, resulting in striking ecological and physiological diversity. Although such associations provide valuable insights into the evolution of symbiosis and adaptation to novel ecological niches, animal–chlorophyte photosymbioses remain relatively understudied. Here, we present an overview of photosymbioses between animals and chlorophytes, highlighting their independent evolutionary origins, ecological diversity and emerging genomic resources. Focusing on Porifera, Cnidaria and Xenacoelomorpha, we review shared and lineage-specific adaptations underlying these associations. We also contrast them with dinoflagellate-based systems to demonstrate their distinct ecological and cellular features. Our work sets the stage for elucidating the molecular mechanisms underlying these associations, enhancing our understanding of how interspecies interactions drive adaptation to unique ecological niches through animal–chlorophyte symbiosis.

## 1. Introduction

Photosynthetic symbiosis, or photosymbiosis, is a close and long-term association between distinct organisms in which an autotrophic endosymbiont resides within a heterotrophic host. These associations involve complex metabolic interactions that can profoundly influence the life history and evolutionary fitness of both partners [1–3]. Photosymbiosis has facilitated the radiation of animals in aquatic environments, as the macronutrients provided by algal photosymbionts enable their hosts to explore and exploit new ecological niches [4–6]. A wide range of animal lineages have photosymbiotic associations with various types of broadly defined ‘algae’, such as green algae, dinoflagellates and diatoms [3]. The multiple evolutionary origins of these associations in both hosts and photosymbionts have resulted in multifaceted ecological and physiological characteristics.

**Figure 1.**
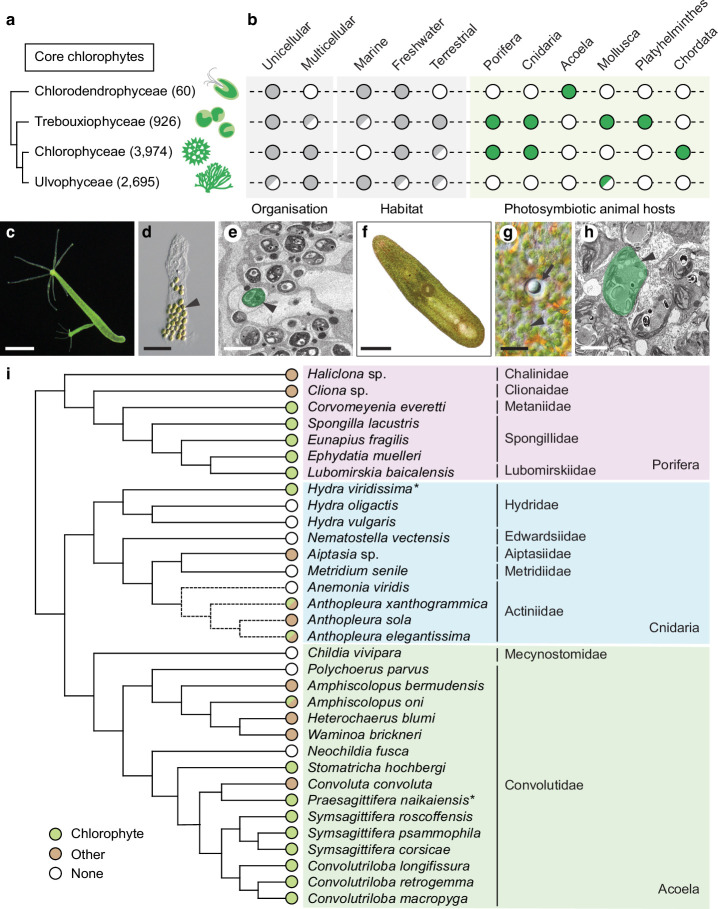
Chlorophyte photosymbiosis across animals. (a) Core chlorophyte classes with species numbers in parentheses. (b) Left and middle: key traits of each class, with filled circles indicating predominant features and half-filled circles indicating less common ones. Right: animal associations with chlorophyte classes; half-filled circles represent kleptoplasty in sacoglossans, where plastids are retained but algae are digested. (c–e) *Hydra–Chlorella* photosymbiosis: (c) *Hydra viridissima* with *Chlorella* (Trebouxiophyceae) (scale bar, 2 mm); (d) host epithelial cell with intracellular symbionts (arrowhead; scale bar, 30 µm); (e) transmission electron microscopy image of endoderm with symbiont (arrowhead; scale bar, 5 µm). (f–h) Acoel–*Tetraselmis* symbiosis: (f) *Praesagittifera naikaiensis* with *Tetraselmis* (Chlorodendrophyceae) (scale bar, 300 µm); (g) anterior view showing statocyst (arrow) and extracellular symbionts (arrowhead; scale bar, 20 µm); (h) transmission electron microscopy image of parenchyma with symbiont (arrowhead; scale bar, 5 µm). Note that the algal symbionts appear irregular in shape due to the loss of their cell walls. (i) Distribution of chlorophyte photosymbiosis in Porifera, Cnidaria and Acoela. Asterisks highlight *H. viridissima* and *P. naikaiensis*, the species shown in the above images. Photosymbiotic states are marked by circle colour: green for chlorophyte photosymbiosis, brown for other types of photosymbiosis and white for no photosymbiosis reported. Phylogeny based on the following studies: Porifera [8,9], Cnidaria [10–12] and Acoela [13,14]. Dashed lines indicate debated monophyletic placements.

The core chlorophytes are a phenotypically rich and ecologically important clade of green algae, falling under the division Chlorophyta—one of the two major clades forming the green plant lineage [7]. Having originated from simple unicellular planktonic marine algae, core chlorophytes diversified drastically in form and radiated into freshwater and terrestrial environments. They now consist of four major classes: Chlorodendrophyceae, unicellular planktonic algae characterized by four flagella; Trebouxiophyceae, a diverse lineage including unicellular coccoid algae and terrestrial lichen algae; Chlorophyceae, the most species-rich class abundant in freshwater habitats; and Ulvophyceae, predominantly benthic marine macroalgae (figure 1a,b) [7,15,16]. These four classes contain all known green algal photosymbionts of animals, and photosymbiosis arose independently in numerous distinct algal lineages. Photosymbiotic species are scattered across Trebouxiophyceae in particular, with especially high representation in the family Chlorellaceae [17,18].

In this review, we summarize the current understanding of chlorophyte photosymbiosis in animals, with a comparative perspective across Porifera, Cnidaria and Xenacoelomorpha—the three phyla in which this trait is best characterized—to highlight its commonality and diversity. We also contrast these associations with well-studied dinoflagellate-based systems, such as those in corals and giant clams. Given the numerous independent origins of this trait across lineages, we discuss ecological factors that may influence the evolvability of animal–chlorophyte photosymbiosis. We highlight key adaptations that facilitate these associations, such as symbiosomes in cnidarians, which are derived from host phagosomes and serve to compartmentalize intracellular symbionts, and phototaxis in acoels, which enables symbiotic animals to optimize light exposure. These are discussed alongside the underlying molecular interactions that support and regulate photosymbiosis. In light of recent advances in omics approaches and the growing availability of genomic data, we provide an overview of current genomic resources for photosymbiotic animals and chlorophytes. Overall, animal–chlorophyte photosymbiosis is characterized by complexity and heterogeneity, indicating the need for further investigation in this underexplored area.

## 2. Evolution of animal–chlorophyte photosymbioses

Chlorophyte photosymbiosis has evolved independently in multiple animal lineages, yet certain species have emerged as key models for studying this association in detail. Recently, the facultatively symbiotic freshwater sponge *Ephydatia muelleri* (Porifera) has received increased attention as a model system [19]. In addition to the sponge, obligate symbioses are exemplified by two well-established systems: the freshwater hydrozoan *Hydra viridissima* (Cnidaria), commonly known as green hydra (figure 1c–e) [20], and the marine acoel *Symsagittifera roscoffensis* (Xenacoelomorpha), a small acoelomate worm inhabiting intertidal zones (figure 1f–h) [2,21]. These three phyla span several major branches of animal evolution: Porifera and Cnidaria are early diverging metazoans, while Xenacoelomorpha is thought to be the sister group to all other Bilateria or to Ambulacraria [22–24]. Despite their independent origins, these systems converge on similar symbiotic traits, such as host-controlled symbiosomes in cnidarians and sponges (figure 1i, table 1 and electronic supplementary material, table S1) [38–40].

**Table 1.** Representative chlorophyte photosymbiotic systems across the animal kingdom.

phylum	class	family	environment	species	cellular position	acquisition	association	chlorophyte class
Porifera	Demospongiae	Spongillidae	freshwater	*Spongilla lacustris* [25]	intracellular	horizontal and vertical	facultative	Trebouxiophyceae, Chlorophyceae
				*Ephydatia muelleri* [26]				
		Lubomirskiidae		*Lubomirskia baicalensis *[27–29]				
Cnidaria	Hydrozoa	Hydridae	freshwater	*Hydra viridissima* [30]	intracellular	vertical	obligate	Trebouxiophyceae
				*Hydra vulgaris* [31–33]		horizontal and vertical	facultative	Chlorophyceae
	Hexacorallia	Actiniidae	marine	*Anthopleura elegantissima *[34]	intracellular	horizontal	facultative	Trebouxiophyceae
				*Anthopleura xanthogrammica *[34]				
Xenacoelomorpha	Acoela	Convolutidae	marine	*Praesagittifera naikaiensis* [35,36]	extracellular	horizontal	obligate	Chlorodendrophyceae
				*Symsagittifera roscoffensis *[2,21,37]				

In Porifera, intracellular photosymbioses have been documented in freshwater sponges of the order Spongillida (table 1). These sponges facultatively establish intracellular associations with green algae from multiple families, including Chlorellaceae, Coccomyxaceae (Trebouxiophyceae) and Mychonastaceae (Chlorophyceae) (figure 1i and table 1) [25,26,28,29]. These sponge associations involve polyphyletic algal partners and vary geographically, reflecting the broader ecological flexibility of the sponge holobiont [25,26]. This pattern suggests that members of Spongillida may possess a generalized capacity for green algal photosymbiosis, with associations forming opportunistically based on local environmental availability.

In Cnidaria, the green hydra *H. viridissima* offers a particularly tractable framework for investigating the dynamics of animal–chlorophyte symbiosis. As the earliest branching member of the genus *Hydra*, it established a stable association with *Chlorella* species (Trebouxiophyceae) following its divergence from other species within the genus (table 1) [10,11,41]. The paraphyly of *Chlorella* symbionts initially suggested that photosymbiosis in *H. viridissima* may have arisen through multiple independent acquisition events [42]. However, molecular phylogenetic analyses have identified five host–symbiont cospeciation events, supporting an alternative scenario in which symbiotic *Chlorella* lineages reverted to a free-living state during an early period of unstable association [17]. These host–symbiont partnerships are maintained across generations via vertical transmission [43,44], and the symbionts reside within host-derived symbiosomes, physically isolated from the external environment (figure 1d,e). This compartmentalization suggests that adaptation to the host niche may have led to a reduction in algal autonomy [20]. By contrast, *H. vulgaris*, commonly known as the brown hydra, rarely harbours symbionts in nature (table 1). Nonetheless, it can sustain artificially introduced *Chlorella*, indicating that algal uptake is not strictly restricted in *Hydra* [31,33], although the establishment of stable, long-term symbiosis appears to require high specificity [45].

All photosymbiotic acoels belong to the family Convolutidae—a derived and morphologically diverse group—and are distributed across two distinct clades [13,46,47]. Within the Convolutidae, one clade harbours intracellular, vertically transmitted dinoflagellates, whereas another forms extracellular associations with chlorophytes that are predominantly acquired through horizontal transmission (figure 1h,i and table 1). Many species within the second clade, including *S. roscoffensis* and *Praesagittifera naikaiensis*, exhibit high specificity for *Tetraselmis* species (Chlorodendrophyceae) and are obligately dependent on their symbionts for survival [48]. Notably, symbiont identity can differ even among sympatric acoel species [49]. Both clades include early branching, non-photosymbiotic taxa, suggesting that photosymbiosis evolved independently in the two lineages. A few species deviate from these general patterns: *Amphiscolops oni* harbours multiple algal symbionts, while *Convoluta convoluta* associates with a diatom (figure 1i and electronic supplementary material, table S1). Together with findings from Porifera and Cnidaria, these observations suggest the evolutionary plasticity of animal–chlorophyte photosymbioses and the diverse strategies through which they have emerged across the animal tree of life.

## 3. Ecological factors underlying photosymbioses

During photosymbiosis, environmental factors such as temperature [50–52], light availability [53,54] and salinity [55,56] have been shown to influence the photosynthetic performance of algal symbionts. In particular, differences in photosynthetic pigments between dinoflagellates and chlorophytes are closely linked to their distinct ecological niches. Dinoflagellates possess chlorophyll *c* and the carotenoid peridinin, which enable absorption of green to yellow wavelengths (approximately 470−550 nm), optimizing light harvesting in clear marine environments where these wavelengths penetrate most effectively [57–60]. In contrast, chlorophytes utilize chlorophyll *b*, which primarily absorbs blue (approx. 455 nm) and red light. Animal–chlorophyte symbioses are typically found in turbid, eutrophic or shallow-water habitats, such as riverbanks, intertidal zones or freshwater lakes, where the underwater light spectrum is altered by suspended particles and dissolved organic matter, resulting in a reduction of red and blue light penetration. In such conditions, chlorophyll *b* may facilitate efficient harvesting of the remaining short-wavelength light near the surface [57,59,61,62]. These distinct light regimes likely contributed to the divergent evolution of light-harvesting pigments in dinoflagellates and chlorophytes. This physiological constraint is reflected in the ecology of chlorophyte-photosymbiotic animals, including hydras, acoels and freshwater sponges, whose distributions are similarly limited to shallow, illuminated and nutrient-rich environments.

**Figure 2.**
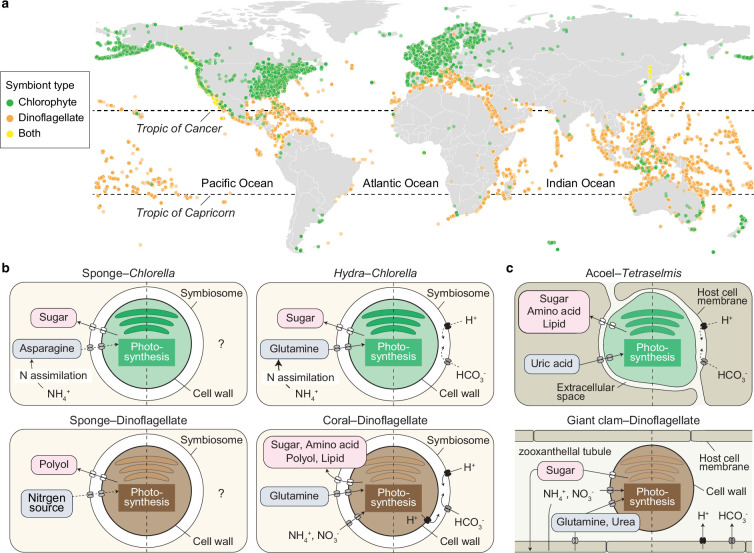
Geographical distribution and cellular organisation of chlorophyte and dinoflagellate photosymbioses in animals. (a) Global distribution of animals hosting chlorophyte or dinoflagellate symbionts, based on records from the Global Biodiversity Information Facility. (b,c) Nutrient exchange between hosts and symbionts in intracellular (b) and extracellular (c) photosymbioses. In acoel–*Tetraselmis* symbioses, loss of the cell wall results in irregular algal cell shape. Each panel illustrates exchanged metabolites (left) and the carbon-concentrating mechanism (right), separated by dashed lines. Note that direct nitrate transfer has not been reported in photosymbioses with chlorophytes. Question marks denote hypothetical mechanisms that have not yet been confirmed. Dashed arrows indicate putative transporters with unknown molecular identity.

The influence of ecological factors becomes evident when comparing the global distributions of animals engaged in green algal versus dinoflagellate photosymbioses. By mapping georeferenced records from the Global Biodiversity Information Facility, we found that animals with green algal symbionts are more prevalent in cooler climates, particularly in regions north of the Tropic of Cancer and south of the Tropic of Capricorn (figure 2a). These species occur in both marine and freshwater environments, typically along coastlines or in inland waters. In contrast, animals that harbour dinoflagellate symbionts, such as corals and giant clams, are primarily concentrated in the warm, clear, coastal waters of the tropics. These patterns suggest that dinoflagellate photosymbiosis is better suited to warmer environments, whereas green algal symbiosis may be favoured under cooler conditions.

This pattern is supported by studies of the sea anemones *Anthopleura elegantissima* and *A. xanthogrammica*, which associate with chlorophyte and/or dinoflagellate photosymbionts (figure 1i and table 1), and regulate the relative abundance of symbiont types in response to environmental conditions [63,64]. Dinoflagellate-rich symbioses are more commonly found at lower latitudes, likely reflecting the higher thermal tolerance of dinoflagellate symbionts [65–67]. Although the cold tolerance of chlorophyte symbionts has not been directly characterized, the ability to associate with multiple algal partners may enhance host fitness by increasing resilience to environmental fluctuations [64,66].

In contrast to poriferans and cnidarians, the motility of photosymbiotic acoels enables them to adapt to their environment and optimize photosynthesis through circatidal vertical migration [68]. Like other bilaterians, acoels possess innervated sensory structures, including photoreceptors and a gravity-sensing organ (figure 1g) [69,70]. These organs enable them to detect environmental cues such as light and gravity, allowing movement to the sand surface or burrowing into the substrate as needed. This behavioural plasticity enables *S. roscoffensis* to regulate light exposure, supporting its photosymbiotic lifestyle in intertidal zones where light intensity, temperature and osmolarity fluctuate markedly throughout the day [71,72]. Together, these examples illustrate how ecological and behavioural traits shape the distribution, stability and evolution of animal–chlorophyte photosymbioses across diverse lineages.

## 4. Molecular basis of photosymbioses

Photosymbioses involve the bidirectional exchange of essential nutrients. Classically, isotopic tracers have been applied to understand metabolic interactions between hosts and symbionts [73–76], since this technique allows us to trace the flow of elements such as carbon and nitrogen. More recently, nanoscale secondary ion mass spectrometry (NanoSIMS) has enabled the ultrastructural mapping of nutrient fate by visualizing the subcellular distribution of elements and their stable isotopes [77–79]. These studies have revealed that hosts receive photosynthetically fixed carbon, such as sugars and lipids, from their algal symbionts, while the symbionts obtain inorganic nitrogen compounds, including nitrate and ammonium, as well as amino acids, from the host [4,74,80–82]. The specific metabolic products exchanged vary across host–symbiont pairings (figure 2b), indicating that each association has evolved distinct metabolic characteristics. In the green hydra *H. viridissima*, the host supplies nitrogen in the form of glutamine [83], and this is reflected in the symbiont’s loss of key genes required for nitrate assimilation, including nitrate transporters and nitrite reductase [20]. This gene loss likely contributes to the reduced autonomy of the symbiont, in contrast to symbiotic dinoflagellates, which retain the capacity to assimilate inorganic nitrogen independently (figure 2b,c) [84]. In the freshwater sponge *E. muelleri*, asparagine synthetase is upregulated in the symbiotic state and may play a key role in nitrogen provisioning (figure 2b) [19]. Corals and giant clams inhabit oligotrophic marine environments, where the ability to assimilate inorganic nitrogen remains important for managing nitrogen availability. By contrast, in nutrient-rich environments such as those inhabited by green hydra, metabolic efficiency may be favoured, with symbionts specializing in carbon fixation while relying on the host for nitrogen [20]. However, the mechanisms of nutrient transport in photosymbiotic acoels and sponges remain poorly understood, although several candidate genes have been identified [19,85].

In addition to nutrient exchange, host mechanisms that enhance symbiont photosynthesis also play a critical role in maintaining photosymbiosis. One such adaptation is the carbon-concentrating mechanism (CCM), which improves photosynthetic efficiency in low-CO_2_ environments [86]. In corals and giant clams, a host-controlled CCM has been identified, with vacuolar-type H^+^-ATPase (VHA) playing a central role [87,88]. VHA, a ubiquitous eukaryotic enzyme, uses energy from ATP hydrolysis to transport protons across biological membranes and localizes to the host membrane surrounding the symbionts (figure 2b,c). This proton transport acidifies the lumen, promoting the conversion of HCO_3_^-^ and H^+^ into CO_2_, which can then diffuse into the algal cells to support photosynthesis. Although there is currently no direct evidence that VHA enhances photosynthesis in chlorophyte symbionts, it has been proposed that VHA may regulate acidification in host-derived symbiosomes in green hydra and in the extracellular environment of acoels (figure 2b,c) [20,85].

The spatial positioning of symbionts within the host is a critical factor shaping molecular interactions in photosymbioses. Electron microscopy has significantly contributed to our understanding of cellular structures, including symbiosomes, and has facilitated the identification of cell types involved in photosymbiosis [37,89,90]. Following the establishment of symbiosis, chlorophyte algae may be maintained intracellularly within host cells, as observed in green hydra and sponges, or extracellularly, as in acoels. In intracellular systems, such as those between corals and their dinoflagellate symbionts from the family Symbiodiniaceae [91], the symbionts are housed within host-derived symbiosomes (figures 1d,e and 2b) [92], whereas in acoels, chlorophyte symbionts are located in the extracellular space within the parenchymal tissue (figures 1h and 2c) [2]. Unlike giant clams, which possess highly specialized mantle tubules known as zooxanthellal tubes for housing symbionts (figure 2c) [93], acoels lack dedicated photosymbiotic organs. A central challenge in photosymbiosis is maintaining metabolic cooperation while preventing cellular damage to both partners. In intracellular systems, specific host cells, such as the endodermal or gastrodermal cells in cnidarians, may facilitate nutrient exchange and regulate host-controlled CCM. However, this close proximity also increases the host’s exposure to reactive oxygen species (ROS) generated during photosynthesis [87]. In contrast, in acoels, ROS produced by extracellular symbionts are less likely to compromise the oxidative status of host cells directly due to spatial separation [94]. Together, these contrasting structural arrangements highlight the diversity of cellular strategies that have evolved to balance metabolic integration and physiological protection in animal–chlorophyte photosymbioses.

## 5. Genomic resources for studying photosymbioses

Understanding photosymbiosis at the molecular level increasingly relies on high-quality genomic and transcriptomic data [85]. Genomes, for instance, enable the identification of genes involved in nutrient exchange, host–symbiont recognition and cellular adaptation. More broadly, they have transformed evolutionary biology by providing insights into phylogeny, gene regulation and cell differentiation [95]. In photosymbiotic systems, comparative host–symbiont genomics has revealed specific metabolic dependencies between partners [20].

**Figure 3.**
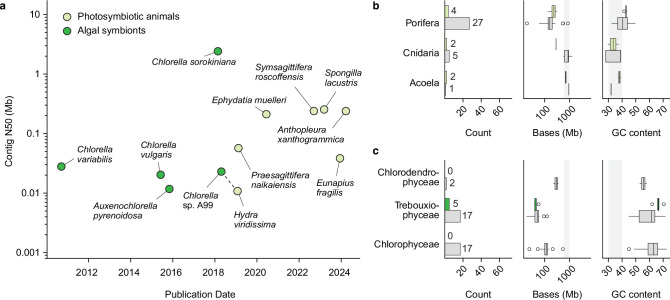
Genomic data availability and characteristics of animal–chlorophyte photosymbiosis. Metadata were retrieved from NCBI Datasets as of May 2024. (a) Genome assembly quality over time, based on contig N50. Assemblies with a contig N50 below 10 kb were excluded. Animal–chlorophyte photosymbiosis pairs are highlighted with dashed lines. (b,c) Genome availability, assembly size and GC content of photosymbiotic animals and closely related non-symbiotic species (b), and of available chlorophyte genomes (c). Photosymbiotic species are shown in green; closely related non-photosymbiotic species are shown in grey. Chlorophyte genomes tend to have higher GC content and smaller genome sizes compared to their animal hosts. A grey-shaded area indicates the typical GC content (30−40%) and genome size (500–1000 Mb) range of animal genomes.

Currently, reference genomes are available for 14 chlorophyte–photosymbiotic animal species across four phyla (figure 3a). Chromosome-level assemblies have been generated for the freshwater sponge *E. muelleri* and the acoel *S. roscoffensis*, both of which are now being used to study the molecular basis of symbiosis. For example, the genome of *E. muelleri* has facilitated the identification of genes related to nitrogen exchange and host–symbiont interactions [19,96]. Further efforts are underway through the Aquatic Symbiosis Genomics (ASG) project, a global initiative launched in 2021 by the Wellcome Sanger Institute, which aims to sequence 1000 high-quality genomes from 500 symbiotic systems [97]. As part of this effort, genomes of the freshwater sponge *Spongilla lacustris*, symbiotic with *Lewiniosphaera* and *Choricystis* species (Trebouxiophyceae), and the sea anemone *Anthopleura xanthogrammica*, symbiotic with *Elliptochloris* species (Trebouxiophyceae), have been released, contributing to a growing genomic framework for investigating animal–chlorophyte photosymbioses (figure 3b,c and table 1) [25,34].

Despite advances in genome sequencing technologies, chromosome-level assemblies are currently available for only a few animal hosts, with their chlorophyte symbionts still lacking equivalent resources. Among chlorophyte photosymbiotic systems, only the pairing of *H. viridissima* and *Chlorella* sp. A99 currently has draft genomic resources available for both partners (figure 3a). Such genomic data are essential for analysing transcriptomes and investigating transcriptional changes in both partners during photosymbiosis. In dinoflagellate-based systems, assembling complete symbiont genomes has been particularly challenging. This is due to their dynamic community composition of Symbiodiniaceae symbionts, which shifts in response to varying environmental conditions [98,99] and their exceptionally large genome sizes, which range from 1 to 250 Gb [100]. In contrast, chlorophyte genomes are much smaller, typically between 20 and 300 Mb, making sequencing and assembly more tractable (figure 3b,c). Furthermore, chlorophyte genomes have a much higher GC content than metazoan genomes. While animal sequences typically have a GC content between 30% and 40%, green algae range from 50% to 70%. This clear, non-overlapping difference suggests distinct gene regulatory mechanisms and provides a useful feature for developing bioinformatics pipelines. The contrasting GC content generates distinct *k*-mer signatures, which can be exploited to distinguish between host and symbiont sequences and to jointly analyse gene expression within the holobiont. These advantages make animal–chlorophyte photosymbioses promising model systems for exploring the genomic basis of symbiosis and host–symbiont co-evolution.

## 6. Conclusion

Animal–chlorophyte photosymbioses have been studied for over a century. A key breakthrough in the laboratory study of these systems has been the ability to maintain hosts and symbionts independently over long periods. This enables researchers to access non-symbiotic states, such as bleached green hydra, aposymbiotic juveniles of acoels and free-living algae [2,20]. These cleaner experimental conditions support the development of genetic resources that are free from contamination, which is essential for identifying lineage-specific features and uncovering traits involved in the emergence of symbiotic mechanisms.

Cellular interactions between hosts and symbionts are central to photosymbiosis. Intracellular and extracellular associations may offer distinct advantages, such as more efficient nutrient transfer or better management of oxidative stress. These interactions affect not only the symbiotic interface but also the broader host tissue context. For instance, photosynthetic products must be redistributed to the appropriate host cells. Classical studies using isotopic tracers and electron microscopy have revealed aspects of nutrient transport and the cell types involved [73–76]. Yet, the genetic and spatial transcriptomic basis of these processes remains poorly understood. Approaches such as single-cell RNA sequencing during symbiosis establishment, and under varying environmental conditions, could reveal the dynamic gene expression landscapes that underlie these interactions [101–103]. Identifying symbiosis-associated cell types and their molecular signatures will also allow for evolutionary comparisons across related species with and without symbionts.

This review has explored the diversity and common features of animal–chlorophyte photosymbioses, focusing on symbiont localization, transmission strategies and molecular interactions. Their repeated occurrence across distantly related lineages indicates strong selective pressures favouring the evolution of these associations. As genomic and transcriptomic resources continue to expand, these systems offer a powerful framework for uncovering how interspecies partnerships drive the evolution of cellular function and ecological adaptation.

## Data Availability

This article has no additional data. Supplementary material is available online [104].
